# The value of dementia care towards the end of life—A contingent valuation study

**DOI:** 10.1002/gps.5259

**Published:** 2020-01-24

**Authors:** Nawaraj Bhattarai, Helen Mason, Ashleigh Kernohan, Marie Poole, Claire Bamford, Louise Robinson, Luke Vale

**Affiliations:** ^1^ Population Health Sciences Institute Newcastle University Newcastle upon Tyne UK; ^2^ Yunus Centre for Social Business and Health Glasgow Caledonian University Glasgow UK

**Keywords:** contingent valuation, dementia, end of life care, palliative care, willingness to pay

## Abstract

**Objectives:**

A dementia nurse specialist (DNS) is expected to improve the quality of care and support to people with dementia nearing, and at, the end of life (EoL) by facilitating some key features of care. The aim of this study was to estimate willingness‐to‐pay (WTP) values from the general public perspective, for the different levels of support that the DNS can provide.

**Methods:**

Contingent valuation methods were used to elicit the maximum WTP for scenarios describing different types of support provided by the DNS for EoL care in dementia. In a general population online survey, 1002 participants aged 18 years or more sampled from the United Kingdom provided valuations. Five scenarios were valued with mean WTP value calculated for each scenario along with the relationship between mean WTP and participant characteristics.

**Results:**

The mean WTP varied across scenarios with higher values for the scenarios offering more features. Participants with some experience of dementia were willing to pay more compared with those with no experience. WTP values were higher for high‐income groups compared with the lowest income level (*P* < .05). There was no evidence to suggest that respondent characteristics such as age, gender, family size, health utility or education status influenced the WTP values.

**Conclusion:**

The general population values the anticipated improvement in dementia care provided by a DNS. This study will help inform judgements on interventions to improve the quality of EoL care.

1

**Key Points**

- The need for quality care and support to dementia patients is well recognised both in the United Kingdom and internationally.
- A potential way of improving care and support to dementia patients nearing the end of life is by having a dementia nurse specialist (DNS).
- The general population value the improvement in dementia care resulting from different levels of support that a DNS can provide.
- People with experience of dementia express higher willingness to pay (WTP) and WTP varies depending on the degree of support offered.

## 2 BACKGROUND

In 2017, there were approximately 50 million people worldwide living with dementia and by 2050, this is set to increase to over 130 million.^1^ In the United Kingdom, it is predicted that there will be over 1 million people with dementia by 2025 if the current age specific prevalence remains stable.^2^ In 2015, the cost of dementia care globally was estimated at $818 billion and is expected to increase to $2 trillion by 2030.^1^ The current estimated annual societal cost of dementia in the United Kingdom is £26.3 billion (at 2012/2013 prices).^2^ With such increases the need to provide good quality care and support for people with dementia whilst demand rises is well recognised both in the United Kingdom and internationally.^3^, ^4^

Dementia is a life limiting illness^5^ and those with dementia nearing the end of life (EoL) have palliative care needs similar to those of cancer patients.^6^ Therefore, the approach to ‘end of life care’ is an important component in the provision of appropriate care to dementia patients.^3^, ^7^, ^8^, ^9^ In the United Kingdom, policy has significantly influenced both the quality of EoL care, via an End of Life Care Strategy^10^ (applicable to all illnesses) and dementia care via a National Dementia Strategy^4^ and Prime Minister's Challenge on Dementia 2020 11 published in 2015 by David Cameron (the then Prime Minister). However, care provided at the EoL to people with dementia remains inconsistent in quality and mostly consensus based.^12^, ^13^, ^14^ To address this, the Supporting Excellence in End of life care in Dementia (SEED) Programme in the United Kingdom was undertaken. Following the MRC framework for complex interventions,^15^ and using a mixed methods approach, the SEED programme developed, via co‐design approaches with key stakeholders, a primary care‐led, intervention to enable community‐based professionals to deliver co‐ordinated and proactive care to people with dementia and their families towards, and at, EoL (https://research.ncl.ac.uk/seed/). The intervention comprised a dementia nurse specialist (DNS), working with primary, secondary and community care teams, providing EoL care focused on seven key areas (see Table 1).^16^, ^17^ These features are key to the design of the DNS, and so understanding the value that is placed on these features should be measured when evaluating the DNS.

**Table 1:** Summary of the seven factors influencing good EoL care for people with dementia^16^

**Undertaking timely planning discussions** to ensure plans are discussed when the person with dementia has capacity and that they are documented and disseminated as appropriate.
**Recognising end of life and providing supportive care** to ensure effective management of key symptoms (eg, pain, anxiety and nausea), and minimise distress by providing comfort in a familiar environment.
**Co‐ordination and continuity of care** includes liaison between day and night staff in services and having established links with local services (eg, hospices), particularly for support out of hours.
**Working effectively with primary care** can be facilitated by having a named liaison person in the practice. For care homes, liaison can be improved by regular routine visits and limiting the number of general practices with which residents are registered.
**Managing hospitalisation** includes avoiding unnecessary admissions by appropriate out‐of‐hours support and documentation of wishes and preferences. It also involves managing admission and discharge effectively where hospitalisation is necessary.
**Continuing care after death** to enable family members to be supported by known members of staff who cared for the person with dementia at the end of life. This continuity of care is valued by family members.
**Valuing staff and on‐going learning** facilitates staff retention and results in a more skilled and knowledgeable workforce. Stable staff teams are more able to detect emotional vulnerability in their colleagues and ensure timely and appropriate support.

As, in the United Kingdom, the health care service is funded from taxation and available to everyone, the views of the public should be reflected in the decisions that are made. There is a need for decisions to be made in the management of dementia due to the increasing prevalence of the disease. This includes those with dementia themselves, carers and the general public as a whole. There has previously been work eliciting the views and perspectives of clinicians and carers^18^, ^19^ but there has been a paucity of evidence regarding the preferences of the general public. This study elicited the values of a representative sample of the general public using the contingent valuation method (CVM) to use in an economic evaluation of the DNS role.

Contingent valuation is a commonly used method in the valuation of non‐market goods (such as environmental interventions)^20^ and is being increasingly used in health care. The CVM involves setting up hypothetical scenarios which describe the proposed intervention and the expected health and non‐health outcomes. Specifically, this takes the form of asking the participant their willingness to pay (WTP) for the intervention through a proposed payment vehicle appropriate in the particular context. This can include out of pocket payments, increases on bills and levies and increases in tax.^21^ In this particular context (a publically funded health care system), a taxation vehicle was used, as this is way of funding health care that a UK population would be familiar with. Participants were asked whether they would be willing to pay an amount to make a DNS available to anyone who may need it. The value that participants may choose to give represents what is known as an opportunity cost, which is the benefit forgone from using a resource for one purpose as opposed to its best alternative use.^22^ This amount volunteered demonstrates the willingness to forgo other personal benefits to gain access to the service thus demonstrating their value for it. In our study, we used this technique to measure the value a representative sample of the general public would place on a DNS, and measure the strength of their preference for such an intervention and the range of features provided.

## 3 METHODS

### 3.1 Study design

The CVM was used to measure the monetary valuation in terms of the WTP^21^, ^23^, ^24^ for the expected improvement in dementia care. In this study, a community perspective was taken, with respondents asked to give their WTP for the SEED intervention to be available through the NHS even though they would not (necessarily) benefit from it themselves. Given this perspective, respondents were asked their WTP in the form of an additional tax per month (as the NHS is funded through taxation) that they would pay for the next 10 years. The 10‐year duration was chosen as a meaningful timescale for respondents and representative of how long a policy intervention might exist before it was redesigned.

**Figure 1:**
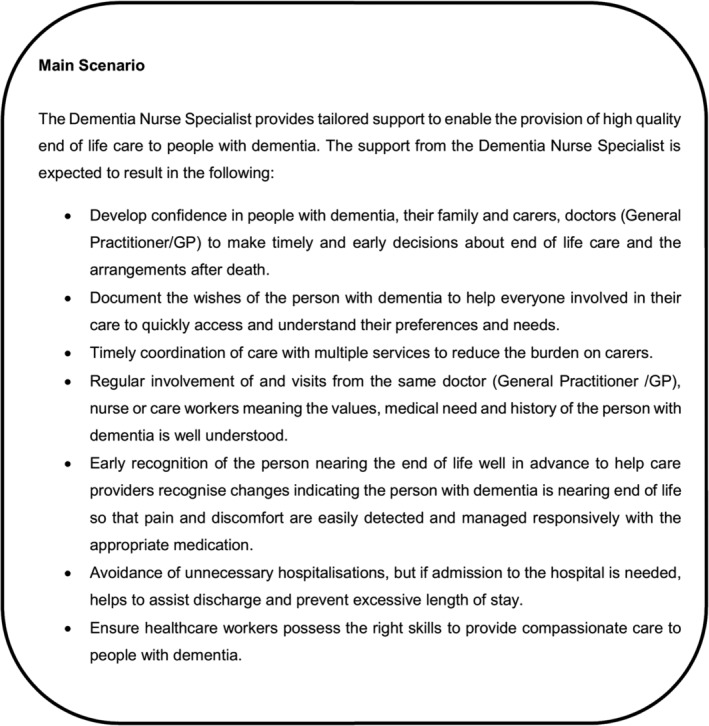
Main scenario

Five scenarios were developed each representing an alternative package of care that could be provided by via the DNS. One scenario had all seven key features of care, the others had a varying number of factors; this was done to assess whether participants valued different features of care differently. The content of the scenarios was based on the seven key components to support good EoL care identified in the SEED intervention (Table 1).^16^ The main scenario is presented in Figure 1, whilst the alternative scenarios used in the study are described in the Supporting Information. The WTP questions formed part of a longer survey that was structured into three sections and delivered online. The first section provided a brief introduction about the disease and its problem, a background on the current practice for dementia care towards the EoL and the DNS intervention. The second section presented the WTP questions. Each respondent was presented with three different scenarios (everyone was presented with the ‘main’ scenario first and then two randomly selected alternatives from the four remaining scenarios). Respondents were presented with a scenario and first asked if they would be willing to pay anything for the intervention as described to be provided. If they answered ‘yes’, then they were presented with a series of payment cards at random on the screen and were asked to state their WTP for the proposed scenario with a question ‘Would you be willing to pay £X for scenario described?’ with ‘X’ representing the randomly picked up amount from the payment cards. Then the respondents were asked to sort out the payment cards by dragging and dropping (using the computer mouse) the WTP amount in the appropriate box (‘Definitely would pay’, ‘Maybe’, ‘Definitely would not pay’) depending on their answers. Twelve levels of monetary amount ranging from £0.50 to £100 were used as payment cards. Generally, four to six levels of monetary payment are considered reasonable.^25^ The respondents were presented with the summary of maximum card value they were definitely willing to pay and the minimum card value they were definitely not willing to pay and were again an open‐ended question to state their maximum WTP within the summarised range of payment values. If the respondents answer ‘No’ to the WTP question on the scenario presented, they were asked to indicate reason for no WTP from a set of reasons or using a free text option. An example of the survey questions is included in the Supporting Information. The third section had questions on respondents' socio‐economic and demographic characteristics such as age, sex, income, education, experience with dementia, current health state (EQ‐5D‐5L and Visual Analogue Scale^26^). Income was used as a categorical variable.

### 3.2 Pre‐test and piloting

Pre‐piloting of the CV component of the survey was conducted in order to test: (a) usability, (b) understanding of the scenarios. The pre‐piloting work was undertaken as ‘think‐aloud’ interviews with seven members of Institute of Health & Society (IHS), Newcastle University, comprising of members of the SEED research team, health economists and administrative staff (with and without experiences of working in dementia research). Piloting of the full web survey was conducted in a sub‐sample of the target general population. The study team considered the pilot sample size (n = 270) was big enough to conduct preliminary analysis. This pilot sample for the general population was recruited from the online panels managed by a marketing company (ResearchNow). Piloting allowed the detection of potential inconsistencies and improvement of clarity. Changes to the categories of zero WTP in the questionnaire were made after the pilot. The pilot participants were not included in the final sample.

### 3.3 Study participants

Whilst earlier CV studies in dementia generally estimated the value placed by sample of carers of people with dementia or service providers, it would be most appropriate to generate the values of people with dementia for whom the interventions are intended.^27^ Therefore, the participants in this study were sampled from the online panel members of the market research company, ResearchNow. The sample was selected to be representative of the UK general public by age (18 years or above) and gender.

There is no formal framework for calculating a sample size for a contingent valuation study. This sample size has been selected as it is judged to be both feasible in terms of time for recruitment as well as a large enough sample for meaningful statistical analysis including appropriate sub‐group analysis. In this study, a sample of 1000 respondents was targeted with quotas on age, gender and geographical regions to be representative of the UK general population.

### 3.4 Survey administration

The survey was delivered online using randomly selected existing panel members from a market research company, ResearchNow. The company converted a paper‐based questionnaire, created by the researchers, into an online survey and offered a small (£1‐2) incentives in the form of shopping vouchers to participants, as per their normal procedures. The online invitation to participate in the survey was sent to its panel members on 9th March 2018 by ResearchNow and the survey stopped accepting new participants once the targeted sample was fulfilled on 14th March 2018. The survey was approved by the Newcastle University Ethics Committee.

### 3.5 Data analysis

The data were analysed in statistical programming language R.^28^ We report the mean and median WTP for each of the five scenarios considered. Any ‘protest responses’ which indicated zero WTP with a reason such as: ‘I don't think I should have to pay for healthcare’ or ‘the government should pay’ were excluded as a conventional practice in WTP studies.^29^ All other reasons for not being willing to pay anything were interpreted as a true zero value and were included in the analysis. In order to reduce the effect on means of extreme upper end WTP responses, means and medians were trimmed by excluding responses from the top 1% of WTP values.^30^ Given the large proportion of zero WTP values and some very high values and skewed (left and right) data expected, standard regression methods such as ordinary least squares would yield biased and inconsistent estimates. In such circumstance, a Tobit model is the preferred alternative^31^, ^32^ and the impact of respondent characteristics (gender, age, income, education, family size and experience of dementia) on WTP values was investigated using this model for the trimmed sample.

## 4 RESULTS

A total of 1002 individuals completed the online survey. Table 2 presents the number of responses per scenario. Table 2 also presents the number of protest responses and the reasons for not being willing to pay anything for each of the scenarios. Protesting participants believe that the NHS should provide the dementia care services in question and they are not willing to pay anything for something that is the state's responsibility. There were 104 protest responses for the Main scenario and the alternative 1, alternative 2, alternative 3 and alternative 4 had 67, 62, 57 and 65 protest responses, respectively (Table 2).

**Table 2:** Initial sample and protest reasons

	Main	Alt1	Alt2	Alt3	Alt4
**Initial sample** (N)	1002	496	506	500	502
**Number of Yes, positive WTP values**	807	335	327	359	324
**Number of No, zero WTP values**	195	161	179	141	178
**Number which are ‘protest zeros’** a	104	67	62	57	65
**Reasons for not WTP for each scenario**					
I do value the improvement in dementia care, but I cannot afford to pay anything for it	62	49	45	54	41
I do not think I should have to pay for health care	94	61	55	54	60
I think the dementia care without the nurse involvement would be satisfactory	19	29	41	19	35
Other (please specify)	20	22	38	14	42

Abbreviation: WTP, willingness to pay.

a Figures include the protest responses from the ‘other’ category of reasons for not being willing to pay.

The characteristics of respondents after removing the protest responses are presented in Table S1 and of those remaining after excluding the top 1% WTP values^30^ are presented in Table S2.

Table 3 reports the mean and median WTP values (the amounts are additional taxation per month) across the scenarios for both the trimmed (ie, excluding the top 1% of WTP values) and untrimmed (ie, without excluding the top 1% of WTP values) datasets. Including all of the data, the mean WTP value for the main scenario was £40.13 (95% CI = 26.25‐54.01). The mean WTP value computed from the untrimmed data set for the alternative scenarios were much higher than the mean WTP value for the main scenario and the very wide 95% CI indicate the presence of very high outlier values. Trimming the top 1% of WTP values, the mean WTP for the main scenario [£24.19 (95% CI = 21.85‐26.52)] was higher compared with the alternatives. The main scenario and alternative 1 had similar median WTP values. The median for both the trimmed and untrimmed data set generally remained the same.

**Table 3:** Mean and median WTP (£ sterling)

	Main	Alt1	Alt2	Alt3	Alt4
Mean WTP (95% CI)	40.13 (26.25, 54.01)	2357.20 (23, 14 006)	257.47 (28, 1391)	810.22 (27, 4700)	2313.69 (22, 13 750)
Mean WTP (95% CI)^a^	24.19 (21.85, 26.52)	18.38 (15.95, 20.82)	16.18 (13.59, 18.76)	18.36 (15.72, 21.00)	16.99 (14.15, 19.83)
Median WTP (95% CI)	10 (10, 15)	10 (7.5, 10)	7.5 (5, 8)	9.25 (7.5, 10)	6 (5, 9)
Median WTP (95% CI)^a^	10 (10, 12.5)	10 (7.5, 10)	7.5 (5, 8)	8 (7.5, 10)	6 (5, 8)

Abbreviation: WTP, willingness to pay.

a Top 1% WTP values removed; figures expressed are additional taxation per month.

Table 4 summarises the mean WTP values by experience of dementia (ie, contact with family, friends or colleagues with dementia). Across all scenarios, individuals with some experience of dementia were willing to pay more for the improved dementia care service compared with those with no experience of dementia. The observed difference in WTP values between individuals with and without experience of dementia was largest in the main scenario [12.12 (95% CI = 7.81‐16.42)]. However, there is no evidence of a statistically significant difference for alternative 2.

**Table 4:** Sub‐group analysis, with and without experience of dementia (£ sterling)

	Mean WTP (95% CI)				
	Main	Alt1	Alt2	Alt3	Alt4
*Dementia experience*	29.26 (25.72, 32.79)	21.87 (18.33, 25.41)	17.21 (13.72, 20.70)	22.15 (18.14, 26.16)	19.99 (15.75, 24.23)
*No dementia experience*	17.14 (14.67, 19.60)	13.32 (10.40, 16.24)	14.79 (10.94, 18.65)	13.25 (10.33, 16.17)	12.41 (9.41, 15.42)
*Difference in mean WTP* a	12.12 (7.81, 16.42) *P* = .0000	8.55 (3.98, 13.12) *P* = .0003	2.42 (−2.76, 7.60) *P* = .36	9.25 (3.95, 13.85) *P* = .0004	7.58 (2.40, 12.76) *P* = .0042

*Note:* Results based on Top 1% WTP excluded from the main data.

Abbreviation: WTP, willingness to pay.

a Dementia experience − No dementia experience.

The results of the regression analysis of WTP values on selected respondent characteristics for each of the scenarios are presented in Table 5. There was no evidence to suggest that patient characteristics such as age, gender, family size, health utility or education status influenced the WTP values. The mean WTP values increased in the main scenario and alternatives 1 to 3, whilst on average it decreased in alternative 4 with the increase in participants' health score (ie, VAS score), however the statistical tests provided no evidence of a difference in the main scenario and alternative 4. Also, there was no evidence to suggest that household income below the £40 000 to £49 999 level influenced the WTP values across all scenarios.

**Table 5:** Regression analysis (excluding the top 1% WTP values)

Covariates	Coeff (SE)				
	Main	Alt1	Alt2	Alt3	Alt4
**Age**	0.16 (0.09)	−0.08 (0.10)	0.14 (0.11)	0.08 (0.1)	−0.16 (0.13)
**Gender Male**	4.45 (2.73)	6.16 (3.20)	1.7 (3.31)	4.08 (3.19)	4.56 (4.08)
**No dementia experience**	−11.65 (2.77)***	−10.71 (3.24)***	1.38 (3.40)	−6.59 (3.25)*	−9.22 (4.11)*
**Family size**	0.58 (0.96)	−0.50 (0.99)	−0.71 (0.85)	−0.65 (1.0)	−1.0 (1.02)
**Health score**	0.11 (0.08)	0.26 (0.10)*	0.21 (0.10)*	0.23 (0.1)*	−0.001 (0.123)
**Utility**	−11.24 (6.95)	−13.28 (8.26)	−10.25 (8.59)	−8.0 (7.66)	−3.90 (10.36)
**Household income**					
Under £10 000					
£10 000‐£19 999	−1.14 (6.25)	−3.78 (7.32)	11.24 (7.7)	−3.27 (7.02)	4.46 (9.52)
£20 000‐£29 999	3.54 (6.20)	−1.23 (7.23)	11.91 (7.5)	−1.33 (6.81)	9.90 (9.58)
£30 000‐£39 999	3.49 (6.20)	−2.25 (7.25)	6.53 (7.56)	3.92 (6.95)	2.68 (9.46)
£40 000‐£49 999	4.15 (6.50)	−2.02 (7.52)	7.33 (8.11)	−1.94 (7.24)	−5.46 (10.14)
£50 000‐£59 999	9.23 (7.09)	7.96 (8.28)	17.98 (8.6)*	3.73 (8.58)	8.7 (10.38)
£60 000‐£69 999	17.83 (8.24)*	8.51 (9.55)	20.04 (10.06)*	1.59 (8.88)	11.80 (13.58)
£70 000‐£79 999	6.68 (8.66)	0.65 (10.18)	27.69 (10.06)**	6.45 (9.62)	8.29 (13.21)
£80 000‐£89 999	13.67 (8.53)	2.90 (9.85)	23.32 (10.10)*	8.15 (8.36)	25.14 (15.75)
£90 000‐£99 999	19.69 (9.80)*	17.87 (11.20)	7.46 (12.41)	2.18 (11.6)	18.39 (13.77)
£100 000‐£149 999	24.82 (9.03)**	17.48 (9.44)	41.97 (1.77)***	49.10 (11.98)***	16.65 (12.1)
£150 000‐£199 999	7.82 (15.34)	9.63 (16.44)	0.89 (21.07)	−22.97 (25.37)	28.64 (22.88)
£200 000‐£499 999	27.29 (19.41)	13.21 (29.95)	71.49 (19.18)***	50.4 (22.04)*	87.92 (26.99)**
£500 000 or more	44.26 (11.76)***	28.43 (12.15)*	12.19 (17.98)	57.55 (13.59)***	22.28 (17.56)
Prefer not to answer	2.11 (7.12)	−9.87 (8.88)	5.17 (8.53)	−4.18 (7.98)	2.09 (10.94)
**Education**					
Incomplete secondary education (Below GC SE/O level)					
Do not want to disclose	0.16 (18.7)	−25.42 (22.42)	5.20 (27.26)	19.09 (19.39)	2.73 (29.26)
Doctorate, Post‐doctorate or equivalent (Higher Degree)	12.61 (9.39)	−6.65 (10.83)	8.11 (12.75)	7.34 (11.06)	2.55 (13.86)
Postgraduate education completed (eg, Masters)	0.009 (7.84)	−2.71 (8.79)	19.37 (10.56)	−0.36 (9.39)	3.85 (11.27)
Secondary education completed (A level or equivalent)	−1.0 (7.55)	−5.35 (8.47)	15.29 (10.15)	−2.86 (8.78)	4.27 (10.96)
Secondary education completed (GCSE/O level/CSE or equivalent)	1.82 (7.47)	−2.35 (8.52)	17.10 (9.83)	1.09 (8.58)	−2.54 (10.92)
Some vocational or technical qualifications	8.79 (13.35)	0.1 (13.85)	29.65 (21.48)	−4.34 (19.98)	19.44 (17.44)
University education completed (first degree, ie, BA, BSc)	4.72 (7.17)	−3.24 (8.22)	11.28 (9.71)	−4.96 (8.51)	2.83 (10.42)
Vocational or technical qualifications completed (eg, HND, NVQ)	4.12 (7.38)	1.69 (8.59)	21.25 (9.77)*	1.50 (8.66)	6.04 (10.74)

Abbreviation: WTP, willingness to pay.

*** *P* < .001; **P < .01; *P < .05.

In the case of the main scenario individuals with a household income of £60 000 to £69 999 were more likely (*P <* .05) to have a higher WTP compared with those with an income under £10 000. The WTP for the main scenario was also higher for the individuals with a household income of £90 000 to £99 999 (*P <* .05), £100 000 to £149 999 (*P <* .01) and £500 000 or more (*P <* .001) compared with those with income less than £10 000.

The WTP value for alternative 1 was higher only in those with a household income of £500 000 or more (*P <* .05) compared with those with an income below £10 000. The WTP value for alternative 2 was higher in individuals with a household income of £50 000 to £59 999 (*P <* .05), £60 000 to £69 999 (*P <* .05), £70 000 to £79 999 (*P <* .01), £80 000 to £89 999 (*P <* .05), £100 000 to £149 000 (*P <* .001) and £200 000 to £499 999 (*P <* .001) compared with those with an income less than £10 000. Whilst, the WTP value for alternative 3 was significantly higher in individuals with an income £100 000 to £149 000 (*P <* .001), £200 000 to £499 999 (*P <* .05) and £500 000 or more (*P <* .001), the WTP for alternative 4 was higher only in individuals with an income £200 000 to £499 999 (*P <* .01) compared with those with an income of less than £10 000.

## 5 DISCUSSION

The results of this CV study indicated that individuals may be willing to sacrifice a considerable amount of money per month for improved EoL care for people with dementia. This highlights the importance and value of improved dementia care services to the general population. Moreover, a higher WTP value for the main scenario compared with the alternatives indicated that respondents valued a broader improvement in dementia care services than less comprehensive services. The sub‐group analysis showed that the amount individuals were willing to sacrifice differed according to their experience of dementia. Compared with individuals with no experience of dementia, individuals with experience of family members, friends or relatives with dementia placed a higher value on the tailored support from the DNS and the provision of high quality EoL care to people with dementia. This shows the importance of improvement in quality of care towards the EoL to those who are affected by dementia.

Our analysis did not demonstrate any relationship between the WTP value placed on the improvement of dementia care services by age of the respondent, gender, household size or the health utility score. This may indicate that the value of quality dementia care towards the EoL is of importance to all irrespective of age, gender or their health status. The EQ‐5D‐VAS score was not associated with the WTP value for the main scenario and alternative 4; nevertheless, there was a significant increase in WTP values with the unit increase in the score in alternatives 2, 3 and 4. This difference in association of VAS score and WTP across scenarios is difficult to interpret but it may be there were elements of those scenarios that resonated with their own health condition. As would be expected (and a test of theoretical validity), individuals with higher ability to pay give higher WTP values.^21^, ^23^ The WTP values were significantly higher for high‐income groups compared with those on the lowest income level which corroborates with economic welfare theory^21^, ^23^; however, there was no evidence of a simple linear relationship between income and WTP values.

In the absence of the revealed preferences, we used a CV survey, a stated preference method to elicit the WTP values for dementia improvement scenarios. The underpinning assumption of a CV study is that people would pay the amount they stated and consider their income when stating their WTP values. This is central to the validity of the responses. Whilst our study results are based on information collected from a large sample selected to represent the UK general population, the findings should be interpreted in the light of some limitations. The CV survey was designed and developed using internationally recognised methodological standards.^21^, ^33^ Pre‐test and piloting of the survey allowed us to refine and simplify the scenarios and questions. Nevertheless, the validity of the responses could have been affected by biases arising out of the construction of this study or by the interpretation and understanding of the scenarios by the respondents, which was beyond our control. The main scenario was presented first in the sequence of three scenarios presented to the respondents, therefore we cannot rule out any potential ordering effects bias in the WTP responses. Although we made an active effort to take a representative sample of general population, using the internet survey panels could have constrained our results by excluding individuals who have not joined the online panels of the survey company used; the characteristics of the individuals who join may be different from those who do not join the online panels. However, our sample was targeted on quotas on age, gender and geographical regions for close representation of the UK general population.

In terms of implications of our findings for practice, the pilot study of the SEED intervention showed that the DNS intervention, with key features including proactive care planning, care co‐ordination and educating and supporting family and professional carers, was feasible and acceptable and integrated easily into existing structures. The DNS model was also highly valued by all ‘users’, that is, professionals, patients and family carers. Given this, the next stage is to conduct a wider evaluation of the potential benefit of the inclusion of a DNS in the health care service. The WTP values estimated in the study reported here could be used to carry out cost‐benefit analysis (CBA) comparing multiple dementia improvement initiatives in terms of net monetary benefits. While the CBA approach is not as typically used in the evaluation of new health care interventions, it is the recommended and most used approach across the rest of UK public sector and provides a clear decision rule to guide and inform NHS decision making.^34^

The next stage is important as recent UK research has revealed that symptom management in people with advanced dementia is still suboptimal with high levels of observed pain and agitation.^35^ Also despite national policy recommending that older people be cared for in their homes, or usual place of care, for as long as possible including up to death, currently nearly 40% of people with dementia in England die in acute hospitals.^36^ In addition to the public placing high value on the newly developed SEED model, evidence shows there is still an urgent need for interventions which improve quality of care in this complex and challenging area of practice.

## 6 CONCLUSION

Dementia care services provided by the DNS towards, and at, the EoL is perceived by the general population as an element with real value in economic terms. The value of dementia care services is generally not influenced by the individual characteristics such as age, gender or their health status. However, these services are highly valued by individuals with some experience of dementia in their close family members, colleagues or relatives and by those in the upper tiers of income. This demonstrates that the general public do value and perceive benefit for providing care to those with dementia. In an area where direct estimation of quality of life is virtually impossible and extensions to quantity of life are not very relevant, this study helps to determine the value placed on aspects of dementia care. The findings have important policy implications for improvement in dementia care provision and may provide valuable insights to decision makers.

## CONFLICT OF INTEREST

None declared.

## Supporting information


**Table S1** Respondent Characteristics (Top 1% WTP values not excluded)
**Table S2**: Respondent Characteristics (Top 1% WTP values excluded)

## Data Availability

The data that support the findings of this study are available in an anonymised version from the corresponding author upon reasonable request.
