# Supplementary material for: The value of dementia care towards the end of life—A contingent valuation study
Source: Int J Geriatr Psychiatry. 2020 Jan 24;35(5):489–97. doi: 10.1002/gps.5259 (PMC7187265; doi:10.1002/gps.5259)
Supplement: Supplementary file 1 — Table S1 Respondent Characteristics (Top 1% WTP values not excluded) Table S2: Respondent Characteristics (Top 1% WTP values excluded) [file GPS-35-489-s001.docx]

**Supporting information file**

**Table 1A. Respondent Characteristics (Top 1% WTP values not excluded)**

| Respondent characteristics | Main (N=897) | Alt1 (N=428) | Alt2 (N=445) | Alt3 (N=442) | Alt4 (N=436) |
| --- | --- | --- | --- | --- | --- |
| **Gender** |  |  |  |  |  |
| Male | 483 (54%) | 201 (47%) | 199 (44.7%) | 208 (47.1%) | 199 (45.6%) |
| Female | 414 (46%) | 227 (53%) | 246 (55.3%) | 234 (52.9%) | 237 (54.4%) |
| **Age-group** |  |  |  |  |  |
| 18-24 | 139 (15.5%) | 64 (15%) | 70 (15.7%) | 60 (13.6%) | 75 (17.2%) |
| 25-34 | 160 (17.8%) | 75 (17.5%) | 80 (18.0%) | 80 (18.1%) | 72 (16.5%) |
| 35-44 | 148 (16.5%) | 58 (13.6%) | 85 (19.1%) | 79 (17.9%) | 68 (15.6%) |
| 45-54 | 155 (17.3%) | 71 (16.6%) | 79 (17.8%) | 71 (16.1%) | 80 (18.3%) |
| 55-64 | 117 (13.0%) | 63 (14.7%) | 47 (10.6%) | 49 (11.1%) | 64 (14.7%) |
| 65+ | 178 (19.8%) | 97 (22.7%) | 84 (18.9%) | 103 (23.3%) | 77 (17.7%) |
| **Marital Status** |  |  |  |  |  |
| Divorced/separated | 56 (6.2%) | 26 (6.1%) | 27 (6.1%) | 25 (5.7%) | 29 (6.7%) |
| Married/living with partner | 571 (63.7%) | 263 (61.4%) | 297 (66.7%) | 282 (63.8%) | 279 (64%) |
| Single | 243 (27.1%) | 125 (29.2%) | 108 (24.3%) | 121 (27.4%) | 117 (26.8%) |
| Widowed | 26 (2.9%) | 14 (3.3%) | 12 (2.7%) | 14 (3.2%) | 10 (2.3%) |
| Do not want to disclose | 1 (0.1%) | 0 (0%) | 1 (0.2%) | 0 (0%) | 1 (0.2) |
| **Employment Status** |  |  |  |  |  |
| Full-time (30 or more hours per week) | 409 (45.6%) | 189 (44.2%) | 207 (46.5%) | 191 (43.2%) | 205 (47%) |
| Contract, Freelance or Temporary Employee | 8 (0.9%) | 4 (0.9%) | 3 (0.7%) | 1 (0.2%) | 7 (1.6%) |
| Full-time Student | 43 (4.8%) | 18 (4.2%) | 22 (4.9%) | 21 (4.8%) | 20 (4.6%) |
| Homemaker | 34 (3.8%) | 15 (3.5%) | 18 (4%) | 14 (3.2%) | 20 (4.6%) |
| Part-time | 105 (11.7%) | 53 (12.4%) | 51 (11.5%) | 47 (10.6%) | 57 (13.1%) |
| Part-time Student (working LESS than 30 hours per week) | 1 (0.1%) | 0 (0%) | 1 (0.2%) | 1 (0.2%) | 0 (0%) |
| Part-time Student (working MORE than 30 hours per week) | 1 (0.1%) | 0 (0%) | 1 (0.2%) | 1 (0.2%) | 0 (0%) |
| Retired | 177 (19.7%) | 96 (22.4%) | 79 (17.8%) | 102 (23.1%) | 75 (17.2%) |
| Self-employed | 48 (5.4%) | 20 (4.7%) | 24 (5.4%) | 23 (5.2%) | 21 (4.8%) |
| Semi-retired | 5 (0.6%) | 3 (0.7%) | 3 (0.7%) | 4 (0.9%) | 2 (0.5%) |
| Stay-at-Home Parent | 12 (1.3%) | 4 (0.9%) | 8 (1.8%) | 8 (1.8%) | 4 (0.9%) |
| Unemployed | 42 (4.7%) | 18 (4.2%) | 22 (4.9%) | 22 (5%) | 19 (4.4%) |
| Do not want to disclose | 1 (0.1%) | 0 (0%) | 1 (0.2%) | 1 (0.2%) | 0 (0%) |
| Other (please specify) | 11 (1.2%) | 8 (1.9%) | 0 (0%) | 0 (0%) | 0 (0%) |
| **Household Income** |  |  |  |  |  |
| Under £10,000 | 64 (7.1%) | 29 (6.8%) | 33 (7.4%) | 37 (8.4%) | 24 (5.5%) |
| £10,000 - £19,999 | 128 (14.3%) | 65 (15.2%) | 55 (12.4%) | 58 (13.1%) | 69 (15.8%) |
| £20,000 - £29,999 | 135 (15.1%) | 64 (15%) | 72 (16.2%) | 72 (16.3%) | 64 (14.7%) |
| £30,000 - £39,999 | 136 (15.2%) | 65 (15.2%) | 70 (15.7%) | 62(14%) | 76 (17.4%) |
| £40,000 - £49,999 | 105 (11.7%) | 53 (12.4%) | 47 (10.6%) | 52 (11.8%) | 49 (11.2%) |
| £50,000 - £59,999 | 69 (7.7%) | 30 (7%) | 36 (8.1%) | 24 (5.4%) | 41 (9.4%) |
| £60,000 - £69,999 | 38 (4.2%) | 18 (4.2%) | 17 (3.8%) | 22 (5%) | 13 (3%) |
| £70,000 - £79,999 | 32 (3.6%) | 15 (3.5%) | 17(3.8%) | 17 (3.8%) | 14 (3.2%) |
| £80,000 - £89,999 | 36 (4%) | 16 (3.7%) | 22 (4.9%) | 28 (6.3%) | 10 (2.3%) |
| £90,000 - £99,999 | 24 (2.7%) | 13 (3%) | 10 (2.2%) | 11(2.5%) | 13 (3%) |
| £100,000 - £149,999 | 28 (3.1%) | 17 (4%) | 11 (2.5%) | 9 (2%) | 19 (4.4%) |
| £150,000 - £199,999 | 7 (0.8%) | 4 (0.9%) | 3 (0.7%) | 2 (0.5%) | 3 (0.7%) |
| £200,000 - £499,999 | 5 (0.6%) | 2 (0.5%) | 3 (0.7%) | 3 (0.7%) | 2 (0.5%) |
| £500,000 or more | 15 (1.7%) | 8 (1.9%) | 6 (1.3%) | 8 (1.8%) | 6 (1.4%) |
| Prefer not to answer | 71 (7.9%) | 28 (6.5%) | 39 (8.8%) | 37 (8.4%) | 32 (7.3%) |
| **Education** |  |  |  |  |  |
| Do not want to disclose | 6 (0.7%) | 4 (0.9%) | 2 (0.4%) | 3 (0.7%) | 3 (0.7%) |
| Doctorate, Post-doctorate or equivalent (Higher Degree) | 37 (4.1%) | 19 (4.4%) | 18 (4%) | 19 (4.3%) | 18 (4.1%) |
| Incomplete Secondary Education (Below GC SE / O Level) | 38 (4.2%) | 19 (4.4%) | 16 (3.6%) | 19 (4.3%) | 19 (4.4%) |
| Postgraduate Education Completed (e.g. Masters) | 111 (12.4%) | 57 (13.3%) | 55 (12.4%) | 51 (11.5%) | 60 (13.8%) |
| Secondary Education Completed (A Level or equivalent) | 142 (15.8%) | 74 (17.3%) | 67 (15.1%) | 67 (15.2%) | 70 (16.1%) |
| Secondary Education Completed (GCSE / O Level / CSE or equivalent) | 130 (14.5%) | 59 (13.8%) | 72 (16.2%) | 69 (15.6%) | 63 (14.4%) |
| Some Vocational or Technical Qualifications | 12 (1.3%) | 8 (1.9%) | 3 (0.7%) | 3 (0.7%) | 8 (1.8%) |
| University Education Completed (First Degree e.g. BA, BSc) | 275 (30.7%) | 126 (29.4%) | 134 (30.1%) | 134 (30.3%) | 129 (29.6%) |
| Vocational or Technical Qualifications Completed (e.g. HND, NVQ) | 146 (16.3%) | 62 (14.5%) | 78 (17.5%) | 77 (17.4%) | 66 (15.1%) |
| **Main Income Earner** |  |  |  |  |  |
| Yes | 606 (67.6%) | 290 (67.8%) | 297 (66.7%) | 297 (67.2%) | 295 (67.7%) |
| No | 284 (31.7%) | 137 (32%) | 143 (32.1%) | 140 (31.7%) | 140 (32.1%) |
| Don’t Know | 7 (0.8%) | 1 (0.2%) | 5 (1.1%) | 5 (1.1%) | 1 (0.2%) |
| **Dementia Experience*** |  |  |  |  |  |
| Family Member | 272 | 135 | 130 | 128 | 141 |
| Friend | 116 | 57 | 64 | 59 | 62 |
| Colleague | 39 | 22 | 16 | 19 | 20 |
| Relative | 177 | 71 | 96 | 95 | 82 |
| Others | 32 | 19 | 14 | 14 | 18 |
| None | 373 | 173 | 187 | 187 | 172 |
| **Household People** (Mean[95%CI]) | 2.69 [2.60;2.79] | 2.66 [2.50;2.82] | 2.78 [2.59;2.97] | 2.67 [2.51;2.82] | 2.76 [2.57;2.95] |
| **Carer** |  |  |  |  |  |
| Yes | 87 (9.7%) | 47 (11%) | 43 (9.7%) | 46 (10.4%) | 46 (10.6%) |
| No | 810 (90.3%) | 381 (89%) | 402 (90.3%) | 396 (89.6%) | 390 (89.4%) |
| **Health Score**  (Mean[95%CI]) | 77.04[75.79;78.29] | 77.08[75.33;78.83] | 76.71[74.88;78.53] | 77.78[76.06;79.50] | 76.10[74.25;77.96] |
| **Utility** (Mean[95%CI]) | 0.82[0.80;0.83] | 0.82 [0.80;0.84] | 0.81 [0.79;0.83] | 0.81 [0.79;0.84] | 0.81 [0.79;0.83] |

*do not add up to 100% because of multiple answers

**Table 2A: Respondent Characteristics (Top 1% WTP values excluded)**

| Respondent characteristics | Main (N=889) | Alt1 (N=424) | Alt2 (N=437) | Alt3 (N=439) | Alt4 (N=432) |
| --- | --- | --- | --- | --- | --- |
| **Gender** |  |  |  |  |  |
| Male | 410 (46.1%) | 199 (46.9%) | 197 (45.1%) | 206 (46.9%) | 196 (45.4%) |
| Female | 479 (53.9%) | 225 (53.1%) | 240 (54.9%) | 233 (53.1%) | 236 (54.6%) |
| **Age-group** |  |  |  |  |  |
| 18-24 | 136 (15.3%) | 64 (15.1%) | 68 (15.6%) | 59 (13.4%) | 73 (16.9%) |
| 25-34 | 157 (17.7%) | 73(17.2%) | 77 (17.6%) | 76 (17.3%) | 71 (16.4%) |
| 35-44 | 149 (16.8%) | 58 (13.7%) | 84 (19.2%) | 80 (18.2%) | 66 (15.3%) |
| 45-54 | 154 (17.3%) | 71 (16.7%) | 80 (18.3%) | 72 (16.4%) | 81 (18.8%) |
| 55-64 | 115 (12.9%) | 61 (14.4%) | 45 (10.3%) | 49 (11.2%) | 64 (14.8%) |
| 65+ | 178 (20%) | 97 (22.9%) | 83 (19%) | 103 (23.5%) | 77 (17.8%) |
| **Marital Status** |  |  |  |  |  |
| Divorced/separated | 56 (6.3%) | 26 (6.1%) | 27 (6.2%) | 25 (5.7%) | 29 (6.7%) |
| Married/living with partner | 564(63.4%) | 259 (61.1%) | 290 (66.4%) | 280 (63.8%) | 275 (63.7%) |
| Single | 242 (27.2%) | 125 (29.5%) | 107 (24.5%) | 120 (27.3%) | 117 (27.1%) |
| Widowed | 26 (2.9%) | 14 (3.3%) | 12 (2.7%) | 14 (3.2%) | 10 (2.3%) |
| Do not want to disclose | 1 (0.1%) | 0 (0%) | 1 (0.2%) | 0 (0%) | 1 (0.2%) |
| **Employment Status** |  |  |  |  |  |
| Full-time (30 or more hours per week) | 404 (45.4%) | 186 (43.9%) | 203 (46.5%) | 187 (42.6%) | 201 (46.5%) |
| Contract, Freelance or Temporary Employee | 8 (0.9%) | 4 (0.9%) | 3 (0.7%) | 1 (0.2%) | 7 (1.6%) |
| Full-time Student | 42 (4.7%) | 18 (4.2%) | 20 (4.6%) | 20 (4.6%) | 20 (4.6%) |
| Homemaker | 34 (3.8%) | 15 (3.5%) | 18 (4.1%) | 15 (3.4%) | 20 (4.6%) |
| Part-time | 106 (11.9%) | 53 (12.5%) | 51 (11.7%) | 47 (10.7%) | 57 (13.2%) |
| Part-time Student (working LESS than 30 hours per week) | 1 (0.1%) | 0 (0%) | 1 (0.2%) | 1 (0.2%) | 0 (0%) |
| Part-time Student (working MORE than 30 hours per week) | 1 (0.1%) | 0 (0%) | 1 (0.2%) | 1 (0.2%) | 0 (0%) |
| Retired | 177 (19.9%) | 96 (22.6%) | 78 (17.8%) | 102 (23.2%) | 75 (17.4%) |
| Self-employed | 45 (5.1%) | 19 (4.5%) | 23 (5.3%) | 24 (5.5%) | 21 (4.9%) |
| Semi-retired | 5 (0.6%) | 3 (0.7%) | 3 (0.7%) | 4 (0.9%) | 2 (0.5%) |
| Stay-at-Home Parent | 12 (1.3%) | 4 (0.9%) | 8 (1.8%) | 8 (1.8%) | 4 (0.9%) |
| Unemployed | 42 (4.7%) | 18 (4.2%) | 22 (5%) | 22 (5%) | 19 (4.4%) |
| Do not want to disclose | 1 (0.1%) | 0 (0%) | 1 (0.2%) | 1 (0.2%) | 0 (0%) |
| Other (please specify) | 11 (1.2%) | 8 (1.9%) | 5 (1.1%) | 6 (1.4%) | 6 (1.4%) |
| **Household Income** |  |  |  |  |  |
| Under £10,000 | 63 (7.1%) | 29 (6.8%) | 33 (7.6%) | 37 (8.4%) | 24 (5.6%) |
| £10,000 - £19,999 | 128 (14.4%) | 65 (15.3%) | 55 (12.6%) | 58 (13.2%) | 69 (16%) |
| £20,000 - £29,999 | 134 (15.1%) | 63 (14.9%) | 71 (16.2%) | 72 (16.4%) | 64 (14.8%) |
| £30,000 - £39,999 | 136 (15.3%) | 64 (15.1%) | 69 (15.8%) | 62 (14.1%) | 75 (17.4%) |
| £40,000 - £49,999 | 105 (11.8%) | 52 (12.3%) | 47 (10.8%) | 51 (11.6%) | 48 (11.1%) |
| £50,000 - £59,999 | 68 (7.6%) | 30 (7.1%) | 35 (8%) | 24 (5.5%) | 40 (9.3%) |
| £60,000 - £69,999 | 38 (4.3%) | 18 (4.2%) | 17 (3.9%) | 22 (5%) | 13 (3%) |
| £70,000 - £79,999 | 32 (3.6%) | 15 (3.5%) | 17 (3.9%) | 17 (3.9%) | 14 (3.2%) |
| £80,000 - £89,999 | 36 (4%) | 16 (3.8%) | 22 (5%) | 28 (6.4%) | 10 (2.3%) |
| £90,000 - £99,999 | 23 (2.6%) | 12 (2.8%) | 10 (2.3%) | 11 (2.5%) | 12 (2.8%) |
| £100,000 - £149,999 | 27 (3%) | 17 (4%) | 11 (2.5%) | 9 (2.1%) | 18 (4.2%) |
| £150,000 - £199,999 | 7 (0.8%) | 4 (0.9%) | 3 (0.7%) | 2 (0.5%) | 3 (0.7%) |
| £200,000 - £499,999 | 4 (0.4%) | 1 (0.2%) | 3 (0.7%) | 2 (0.5%) | 2 (0.5%) |
| £500,000 or more | 13 (1.5%) | 8 (1.9%) | 4 (0.9%) | 6 (1.4%) | 6 (1.4%) |
| Prefer not to answer | 70 (7.9%) | 28 (6.6%) | 37 (8.5%) | 36 (8.2%) | 32 (7.4%) |
| **Education** |  |  |  |  |  |
| Do not want to disclose | 6 (0.7%) | 4 (0.9%) | 2 (0.5%) | 3 (0.7%) | 3 (0.7%) |
| Doctorate, Post-doctorate or equivalent (Higher Degree) | 37 (4.2%) | 17 (4%) | 17 (3.9%) | 19 (4.3%) | 17 (3.9%) |
| Incomplete Secondary Education (Below GC SE / O Level) | 38 (4.3%) | 18 (4.2%) | 17 (3.9%) | 19 (4.3%) | 18 (4.2%) |
| Postgraduate Education Completed (e.g. Masters) | 108 (12.1%) | 57 (13.4%) | 52 (11.9%) | 49 (11.2%) | 57 (13.2%) |
| Secondary Education Completed (A Level or equivalent) | 141 (15.9%) | 74 (17.5%) | 66 (15.1%) | 67 (15.3%) | 70 (16.2%) |
| Secondary Education Completed (GCSE / O Level / CSE or equivalent) | 128 (14.4%) | 59 (13.9%) | 73 (16.7%) | 69 (15.7%) | 64 (14.8%) |
| Some Vocational or Technical Qualifications | 12 (1.3%) | 8 (1.9%) | 3 (0.7%) | 3 (0.7%) | 8 (1.9%) |
| University Education Completed (First Degree e.g. BA, BSc) | 274 (30.8%) | 126 (29.7%) | 130 (29.7%) | 134 (30.5%) | 128 (29.6%) |
| Vocational or Technical Qualifications Completed (e.g. HND, NVQ) | 145 (16.3%) | 61(14.4%) | 77 (17.6%) | 76 (17.3%) | 67 (15.5%) |
| **Main Income Earner** |  |  |  |  |  |
| Yes | 602 (67.7%) | 285 (67.2%) | 293 (67%) | 295 (67.2%) | 292 (67.6%) |
| No | 281 (31.6%) | 137 (32.3%) | 140 (32%) | 140 (31.9%) | 139 (32.2%) |
| Don’t Know | 6 (0.7%) | 2 (0.5%) | 4 (0.9%) | 4 (0.9%) | 1 (0.2%) |
| **Dementia Experience*** |  |  |  |  |  |
| Family Member | 264 | 132 | 123 | 124 | 138 |
| Friend | 112 | 55 | 62 | 55 | 60 |
| Colleague | 36 | 20 | 14 | 17 | 19 |
| Relative | 173 | 69 | 93 | 93 | 81 |
| Others | 32 | 19 | 14 | 14 | 18 |
| None | 372 | 173 | 187 | 187 | 171 |
| **Household People** (Mean[95%CI]) | 2.69[2.59;2.79] | 2.67[2.51;2.83] | 2.76[2.58;2.95] | 2.66[2.50;2.81] | 2.76[2.57;2.95] |
| **Carer** |  |  |  |  |  |
| Yes | 82 (9.2%) | 44 (10.4%) | 40 (9.2%) | 43 (9.8%) | 43 (10%) |
| No | 807 (90.8%) | 380 (89.6%) | 397 (90.8%) | 396 (90.2%) | 389 (90%) |
| **Health Score** | 76.90[75.64;78.16] | 77[75.23;78.76] | 76.75[74.92;78.57] | 77.71[75.99;79.43] | 75.99[74.12;77.85] |
| (Mean[95%CI]) |  |  |  |  |  |
| **Utility** (Mean[95%CI]) | 0.82[0.80;0.83] | 0.82[0.80;0.84] | 0.81[0.79;0.84] | 0.82[0.80;0.84] | 0.81[0.79;0.83] |

**Alternative Scenarios used in the survey**

**Alternative 1**

The Dementia Nurse Specialist provides tailored support to enable the provision of high quality end of life care to people with dementia. The support from Dementia Nurse Specialist is expected to result in the following:

- Timely coordination of care with multiple services to reduce burden on carers.

**Alternative 2**

The Dementia Nurse Specialist provides tailored support to enable the provision of high quality end of life care to people with dementia. The support from Dementia Nurse Specialist is expected to result in the following:

- Develop confidence in people with dementia, their family and carers and doctors (General Practitioner/GP) to make timely and early decisions about end of life care and the arrangements after death.
- Document the wishes of the person with dementia to help everyone involved in their care to quickly access and understand their preferences and needs.

**Alternative 3**

The Dementia Nurse Specialist provides tailored support to enable the provision of high quality end of life care to people with dementia. The support from Dementia Nurse Specialist is expected to result in the following:

- Regular involvement of and visits from the same doctor (General Practitioner /GP), nurse or care workers meaning the values, medical need and history of the person with dementia is well understood.
- Early recognition of the person nearing the end of life well in advance to help care providers recognise changes indicating the person with dementia is nearing end of life so that pain and discomfort are easily detected and managed responsively with the appropriate medication.
- Avoidance of unnecessary hospitalisations, but if admission to the hospital is needed, helps to assist discharge and prevent excessive length of stay.

**Alternative 4**

The Dementia Nurse Specialist provides tailored support to enable the provision of high quality end of life care to people with dementia. The support from Dementia Nurse Specialist is expected to result in the following:

- Ensure healthcare workers possess the right skills to provide compassionate care to people with dementia.

**Questionnaire SAMPLE (***has online programming codes included***)**

**UPN: 44391160**

**Wave: 1**

**SURVEY NAME: Dementia Care**

**SURVEY LENGTH (MINS): 15**

**TYPE OF PROJECT: Full Service Project – specify with or without resume**

**SAMPLE SOURCE/COUNTRY/DETAILS:**

| **COUNTRY** | **LANGUAGE(S)** | **SOURCE(S)** |
| --- | --- | --- |
| **UK** | **English** | **VOP/EROP/EROP NEC/NEC CAN/PL** |

| **REQUIREMENTS** | **QUESTION NUMBERS/ NOTE** |
| --- | --- |
| **OPEN-ENDS (CHA)** |  |
| **PERSONAL IDENTIFIABLE INFO** | **NO** |
| **DEVICES ALLOWED** | **ALL** |
| [Mobile Calculator](http://survey-na.researchnow.com/wix/p980783683.aspx?src=98&C=1) **Score** |  |
| **ADVANCED IMAGE/VIDEO SECURITY** | **NO** |
| **CONJOINT** | **N/A** |
| **ALGORITHM** | **N/A** |
| **MAX DIFF** | **N/A** |
| **OTHER COMPLEX SET UP** | **N/A** |
| **AUTO SPEEDER CHECKS** | **ENABLE** |

**ONLINE REPORTING LINK REQUIREMENTS: AS QUOTAS BELOW IF NOT SPECIFIED**

**QUOTAS: AS PER QUOTA SHEET**

**SURVEY QUESTIONNAIRE**

This survey is being carried out by researchers at the Institute of Health & Society, Newcastle University. We want to know your views on the care provided to people with dementia at the end of life.

The questionnaire has three sections: **PART-A, PART-B** and **PART-C**. It should take no longer than 15 minutes to complete.

- **PART-A** presents you with background information on dementia care at the end of life and the different options for how care can be provided to people with dementia at the end of their life.
- **PART-B** asks questions about the value you place on the different options for care.
- **PART-C** asks general questions about yourself.

There are no right or wrong answers. We are just interested in your views. The questionnaire is anonymous and we will not be asking any personal identifiable information. Your participation is voluntary and you can withdraw from this survey at any time you wish to without giving any reason whatsoever.

This study was approved by the Faculty of Medical Sciences Research Ethics Committee, part of Newcastle University's Research Ethics Committee. This committee contains members who are internal to the Faculty, as well as one external member. This study was reviewed by members of the committee, who must provide impartial advice and avoid significant conflicts of interests.

**[PN - new screen – hold next button for 3 seconds]**

**For this survey our client at the Institute of Health & Society, Newcastle University would like to ask your opinion about a personal or sensitive topic, please be assured that all the answers will be treated as confidential and will be used for research purposes.**

**All data will be processed in adherence to Market Research Society’s Code of Conduct and Data Protection Act 1998.**

1. What is your gender?
   1. Male
   2. Female
2. What is your age? [OENUM OR DROP DOWN]

**[DAGE.1.** **Punch answers from Q2 into age NET]**

1. <18 **[TERMINATE]**
2. 18 – 24
3. 25 - 34
4. 35 - 44
5. 45 - 54
6. 55 - 64
7. 65+
8. In which region do you live?
9. North East

2. North West

3. Yorkshire

4. Scotland

5. Northern Ireland

6. East Midlands

7. West Midlands

8. Wales

9. East

10. London

11. South East

12. South West

**[DREGION. Punch answers from Q3 into regional NET]**

1. North
   1. North East
   2. North West
   3. Yorkshire
   4. Scotland
   5. Northern Ireland
2. Midlands
3. East Midlands
4. West Midlands
5. Wales
6. South
7. East
8. London
9. South East
10. South West

**[PN – show each infox1-4 page on a separate screen. Hold next button for 3 seconds per screen]**

**Infox1**

**Please take your time to read the descriptions below…**

**PART-A**

This part of the survey provides brief description about dementia, the reason for conducting this survey, and a practice question showing what the survey involves.

***What is Dementia?***

The word ‘dementia’ describes a set of symptoms that may include memory loss and difficulties with thinking, problem-solving or language. These changes are often small to start with, but for someone with dementia they have become severe enough to affect daily life. A person with dementia may also experience changes in their mood or behaviour. Dementia is caused when the brain is damaged by diseases, such as Alzheimer’s disease or a series of strokes. Alzheimer’s disease is the most common cause of dementia, but not the only one. The type of symptoms that someone with dementia experiences will depend on the parts of the brain that are damaged and the disease that is causing the dementia.

**Infox2**

***What is the problem?***

People with advanced dementia can receive poorer quality end of life care compared to those with conditions such as cancer, because it can be harder to identify when symptoms are getting worse and the individuals affected with advanced dementia may not be able to say when they are in pain. The person with dementia may also suffer from other conditions that are caused by or which are made worse by dementia.

As a consequence, people with dementia are less likely to die in their preferred place of care and may experience unnecessary hospital admissions in the last year of life. An increasing number of people with dementia are taken to hospital as an emergency admission. A large proportion of those admissions are avoidable.

**Infox3**

***Why this survey is being carried out?***

There are a number of ways the health and social care systems can help people with dementia and their family, and friends at the end of life. We are interested in your views about how we might provide care to people with dementia. The views of the public are important as it is the general public who fund the NHS and are potential users of the services provided.

In this study what the researchers are looking at is one particular way of assessing how important the public think the different options for providing care to people with dementia are compared to each other and how valuable each option is to them.

[Programming Instruction: Could start Next Screen Here]

The NHS is paid for by taxation therefore we would like to know how much people would be willing to pay for each of the different options of care in the form of extra taxation which would be earmarked to provide this care.

We want you to think about how much you would be willing to contribute (not how much you think it would cost), for the NHS to provide each option described. The amount you are willing to pay tells us how important the different care options are to you.

The information is not and will not be used to think about introducing payments for health care but it will help the NHS decide how best to spend the funds available to it.

**Infox4 x**

**To start, we will work through a practice question.**

Imagine you are on holiday abroad in quite a remote place and you get a bad sore throat – bad enough that it is painful every time you swallow food. If you don’t take anything for it, suppose it will last for 3 days. However, someone can arrange to have medicine delivered to you that will cure your sore throat within 24 hours, thereby saving you 2 of the 3 days of painful sore throat. Think about what would be the MOST you would be willing to pay from your household budget to get the medicine delivered.

4. Would you be willing to contribute something, extra from your available household budget, to get this medicine delivered which will reduce your sore throat from 3 days to 1 day? (Please select your answer)

- Yes
- No

**[Ask if Q4=1]**

5. You said you would be willing to contribute for the medicine to be delivered.

What is the maximum you would be willing to contribute for the medicine to be delivered?

When you are thinking about this, please bear in mind what you really think you would or would not be prepared to pay, given your actual income and savings.

To help you decide the amount you would be willing to contribute, you are going to see different amounts of money. For each amount decide if you ‘Definitely Would Pay’, ‘Definitely Would Not Pay’ or ‘Maybe’.

Would you be willing to pay **[pipe in amount from a list below]** for the scenario described earlier?

| **Definitely Would Pay** | **Maybe** | **Definitely Would Not Pay** |
| --- | --- | --- |
|  |  |  |

**[PN - Randomly select. drag and drop buckets. Keep amount visible once in bucket. Respondents should be able to change their answer and drag into another bucket once placed.**

**Validate (and show error) so there shouldn’t be an amount in ‘Definitely would not pay’ that is lower than an amount in ‘Definitely would pay’]**

**[Amount to pipe into question text] [Programming Instruction: The money amounts must appear one at a time in a random order. We do not want the participants to see all of the values at the beginning]**

- £0.50
- £1
- £1.50
- £2
- £5
- £7.50
- £10
- £15
- £20
- £30
- £50
- £100

6. The highest amount you said you WOULD be willing to pay was **[insert highest amount from ‘definitely would pay’ at Q5]**

The lowest amount you said you WOULD NOT be willing to pay was **[insert lowest amount from ‘definitely would not pay’ at Q5].**

What is the **maximum** amount you would be willing to pay, it could be one of these two amounts or something in between.

Maximum willingness to pay: £ **[OENUM text box]**

**[PN - If a respondent has no payment cards in the ‘Definitely Would Not Pay’ column then they should see the following text instead of that above].**

The highest amount you said you WOULD be willing to pay was **[insert highest amount from ‘definitely would pay’ at Q5]**.

What is the MAXIMUM amount you would be willing to pay? It could be this amount or something higher than this.

Maximum willingness to pay: £ **[OENUM text box]**

**[Ask if Q4=2. SC]**

7. You said you would not be willing to pay anything to get the medicine delivered. Why are you not willing to pay anything for this new medicine? Please select **one reason** below:

1. The illness only lasts for 3 days so it is not worth paying for the treatment
2. The symptoms of the illness are not too bad, I could live with it
3. I cannot afford to spend money on medical treatments
4. I don’t think I should have to pay for healthcare
5. Other (please specify) **[OE text box] [Programming Instructions: Can the system read what people enter? Currently if they just write a single alphabet they can move on, but we want this to have a minimum character limit (say 10) and maximum character limit (say 500).**

**Infox5**

**We will now move onto our questions about dementia care in PART B.**

**PART B**

To help people with dementia at the end of life a new service provided by a Dementia Nurse Specialist is being considered by your NHS. You will be shown three different options the Dementia Nurse Specialist service could provide. We would like to know how valuable each of these options would be to you.

**Infox6 [Add a time stamp and hold next button for 3 seconds]**

**Scenario A**

The Dementia Nurse Specialist provides tailored support to enable the provision of high quality end of life care to people with dementia. The support from the Dementia Nurse Specialist is expected to result in the following:

- Develop confidence in people with dementia, their family and carers, doctors (General Practitioner/GP) to make timely and early decisions about end of life care and the arrangements after death.
- Document the wishes of the person with dementia to help everyone involved in their care to quickly access and understand their preferences and needs.
- Timely coordination of care with multiple services to reduce the burden on carers.
- Regular involvement of and visits from the same doctor (General Practitioner /GP), nurse or care workers meaning the values, medical need and history of the person with dementia is well understood.
- Early recognition of the person nearing the end of life well in advance to help care providers recognise changes indicating the person with dementia is nearing end of life so that pain and discomfort are easily detected and managed responsively with the appropriate medication.
- Avoidance of unnecessary hospitalisations, but if admission to the hospital is needed, helps to assist discharge and prevent excessive length of stay.
- Ensure healthcare workers possess the right skills to provide compassionate care to people with dementia.

8. Would you be willing to pay something to have a dementia care service in the NHS as described in the Scenario A? (*Please select your answer below*).

- Yes
- No

**[Ask if Q8=1]**

9. You said you would be willing to contribute through increased taxation which will be earmarked for the dementia care service as described in Scenario A.

What is the maximum you would be willing to contribute each month for the next 10 years for the dementia care service described in Scenario A?

When you are thinking about this, please bear in mind what you really think you would or would not be prepared to pay, given your actual income and savings.

To help you decide the amount you would be willing to contribute for, you are going to see different amounts of money. For each amount decide if you ‘Definitely Would Pay’, ‘Definitely Would Not Pay’ or ‘Maybe’.

Would you be willing to pay **[pipe in amount from a list below]** in taxation per month for the next 10 years for the dementia care service as described in Scenario A? [Programming Instructions: Could you please place this question like this, it is not done this way in the latest link]

| **Definitely Would Pay** | **Maybe** | **Definitely Would Not Pay** |
| --- | --- | --- |
|  |  |  |

**[PN - Randomly select. drag and drop buckets. Keep amount visible once in bucket. Respondents should be able to change their answer and drag into another bucket once placed.**

**Validate (and show error) so there shouldn’t be an amount in ‘Definitely would not pay’ that is lower than an amount in ‘Definitely would pay’]**

**[Amount to pipe into question text]** **[Programming Instruction: The money amounts must appear one at a time in a random order. We do not want the participants to see all of the values at the beginning]**

- £0.50
- £1
- £1.50
- £2
- £5
- £7.50
- £10
- £15
- £20
- £30
- £50
- £100

**[Programming Instructions: If the respondent does not place a figure in the box, a message prompting them to do so should appear]**

10. The highest amount you said you WOULD be willing to pay was **[insert highest amount from ‘definitely would pay’ at Q9]**

The lowest amount you said you WOULD NOT be willing to pay was **[insert lowest amount from ‘definitely would not pay’ at Q9].**

What is the MAXIMUM amount you would be willing to pay from your household budget? It could be one of these amounts or something in between.

Maximum willingness to pay: £ **[OENUM text box]**

**[PN - If a respondent has no payment cards in the ‘Definitely Would Not Pay’ column then they should see the following text instead of that above].**

The highest amount you said you WOULD be willing to pay was **[insert highest amount from ‘definitely would pay’ at Q9]**.

What is the MAXIMUM amount you would be willing to pay? It could be this amount or something higher than this.

Maximum willingness to pay: £ **[OENUM text box]**

**[Ask if Q8=2. SC]**

11. You said you would not be willing to pay anything for the dementia care service described in Scenario A. Why are you not willing to pay anything? Please select **one reason** below:

1. I think the dementia care without the nurse involvement would be satisfactory
2. I do value the improvement in dementia care, but I cannot afford to pay anything for it
3. Other (please specify) [Programming Instructions: Can the system read what people enter? Currently if they just write a single alphabet they can move on, but we want this to have a minimum character limit (say 10) and maximum character limit (say 500).]

**Scenario B**

**[Assign one scenario on a leastfull from the below or alternatives 3-4 in at end of questionnaire. Hold next button for 3 seconds. Add a respondent time stamp at each alternative]**

**Alternative.1**

The Dementia Nurse Specialist provides tailored support to enable the provision of high quality end of life care to people with dementia. The support from Dementia Nurse Specialist is expected to result in the following:

- Timely coordination of care with multiple services to reduce burden on carers.

12. Would you be willing to contribute something through increased taxation which will be earmarked for the dementia care service described in the Scenario B (Please select your answer below).

- Yes
- No

**[Ask if Q12=1]**

13. You said you would be willing to contribute something through increased taxation which will be earmarked for the dementia care service described in Scenario B.

What is the maximum you would be willing to contribute each month for the next 10 years for the dementia care service described in Scenario B?

When you are thinking about this, please bear in mind what you really think you would or would not be prepared to pay, given your actual income and savings.

To help you decide the amount you would be willing to contribute, you are going to see different amounts of money. For each amount decide if you ‘Definitely Would Pay’, ‘Definitely Would Not Pay’ or ‘Maybe’.

Would you be willing to pay **[pipe in amount from a list below]** in taxation per month for the next 10 years for the dementia care service as described in Scenario B? [Programming Instructions: Could you please this question like this, it is not done this way in the latest link]

| **Definitely Would Pay** | **Maybe** | **Definitely Would Not Pay** |
| --- | --- | --- |
|  |  |  |

**[PN - Randomly select. drag and drop buckets. Keep amount visible once in bucket. Respondents should be able to change their answer and drag into another bucket once placed.**

**Validate (and show error) so there shouldn’t be an amount in ‘Definitely would not pay’ that is lower than an amount in ‘Definitely would pay’]**

**[Amount to pipe into question text]** **[Programming Instruction: The money amounts must appear one at a time in a random order. We do not want the participants to see all of the values at the beginning]**

- £0.50
- £1
- £1.50
- £2
- £5
- £7.50
- £10
- £15
- £20
- £30
- £50
- £100

**[Programming Instructions: If the respondent does not place a figure in the box, a message prompting them to do so should appear]**

14. The highest amount you said you WOULD be willing to pay was **[insert highest amount from ‘definitely would pay’ at Q13]**

The lowest amount you said you WOULD NOT be willing to pay was **[insert lowest amount from ‘definitely would not pay’ at Q13].**

What is the **MAXIMUM** amount you would be willing to pay? It could be one of these amounts or something in between.

Maximum willingness to pay: £ **[OENUM text box]**

**[PN - If a respondent has no payment cards in the ‘Definitely Would Not Pay’ column then they should see the following text instead of that above].**

The highest amount you said you WOULD be willing to pay was **[insert highest amount from ‘definitely would pay’ at Q13]**.

What is the **MAXIMUM** amount you would be willing to pay? It could be this amount or something higher than this.

Maximum willingness to pay: £ **[OENUM text box]**

**[Ask if Q12=2. SC]**

**15.** You said you would not be willing to pay anything for the dementia care service described in Scenario B. Why are you not willing to pay anything? Please select one reason below**:**

1. I think the dementia care without the nurse involvement would be satisfactory
2. I do value the improvement in dementia care, but I cannot afford to pay anything for it
3. Other (please specify) [Programming Instructions: Can the system read what people enter? Currently if they just write a single alphabet they can move on, but we want this to have a minimum character limit (say 10) and maximum character limit (say 500).]

**[Alternative 2]**

**Scenario C**

**[Assign one scenario on a leastfull from the below or alternatives 3-4 in at end of questionnaire. Hold next button for 3 seconds. Add a respondent time stamp at each alternative]**

**Alternative.1**

The Dementia Nurse Specialist provides tailored support to enable the provision of high quality end of life care to people with dementia. The support from Dementia Nurse Specialist is expected to result in the following:

The Dementia Nurse Specialist provides tailored support to enable the provision of high quality end of life care to people with dementia. The support from Dementia Nurse Specialist is expected to result in the following:

- Develop confidence in people with dementia, their family and carers and doctors (General Practitioner/GP) to make timely and early decisions about end of life care and the arrangements after death.
- Document the wishes of the person with dementia to help everyone involved in their care to quickly access and understand their preferences and needs.

12. Would you be willing to contribute something through increased taxation which will be earmarked for the dementia care service described in the Scenario C (Please select your answer below).

Yes

No

**[Ask if Q12=1]**

13. You said you would be willing to ~~pay~~ contribute something through increased taxation which will be earmarked for the dementia care service described in Scenario B.

What is the maximum you would be willing to contribute each month for the next 10 years for the dementia care service described in Scenario B?

When you are thinking about this, please bear in mind what you really think you would or would not be prepared to pay, given your actual income and savings.

To help you decide the amount you would be willing to contribute, you are going to see different amounts of money. For each amount decide if you ‘Definitely Would Pay’, ‘Definitely Would Not Pay’ or ‘Maybe’.

Would you be willing to pay **[pipe in amount from a list below]** in taxation per month for the next 10 years for the dementia care service as described in Scenario B?

| **Definitely Would Pay** | **Maybe** | **Definitely Would Not Pay** |
| --- | --- | --- |
|  |  |  |

**[PN - Randomly select. drag and drop buckets. Keep amount visible once in bucket. Respondents should be able to change their answer and drag into another bucket once placed.**

**Validate (and show error) so there shouldn’t be an amount in ‘Definitely would not pay’ that is lower than an amount in ‘Definitely would pay’]**

**[Amount to pipe into question text]** **[Programming Instruction: The money amounts must appear one at a time in a random order. We do not want the participants to see all of the values at the beginning]**

- £0.50
- £1
- £1.50
- £2
- £5
- £7.50
- £10
- £15
- £20
- £30
- £50
- £100

**[Programming Instructions: If the respondent does not place a figure in the box, a message prompting them to do so should appear]**

14. The highest amount you said you WOULD be willing to pay was **[insert highest amount from ‘definitely would pay’ at Q13]**

The lowest amount you said you WOULD NOT be willing to pay was **[insert lowest amount from ‘definitely would not pay’ at Q13].**

What is the **MAXIMUM** amount you would be willing to pay? It could be one of these amounts or something in between.

Maximum willingness to pay: £ **[OENUM text box]**

**[PN - If a respondent has no payment cards in the ‘Definitely Would Not Pay’ column then they should see the following text instead of that above].**

The highest amount you said you WOULD be willing to pay was **[insert highest amount from ‘definitely would pay’ at Q13]**.

What is the **MAXIMUM** amount you would be willing to pay? It could be this amount or something higher than this.

Maximum willingness to pay: £ **[OENUM text box]**

**[Ask if Q12=2. SC]**

**15.** You said you would not be willing to pay anything for the dementia care service described in Scenario C. Why are you not willing to pay anything? Please select one reason below**:**

1. I think the dementia care without the nurse involvement would be satisfactory
2. I do value the improvement in dementia care, but I cannot afford to pay anything for it
3. Other (please specify) [Programming Instructions: Can the system read what people enter? Currently if they just write a single alphabet they can move on, but we want this to have a minimum character limit (say 10) and maximum character limit (say 500).]

**Thank you for completing this section of the survey.**

**Infox7**

**PART- C**

In this final section we would like to collect some general information about you. These questions allow us to ensure we survey a wide range of people with different characteristics. This information will not be connected with your identifiable details.

**16.** What is your current marital status? (*Please select one that applies to you*)

1. Single
2. Married/living with partner
3. Divorced/separated
4. Widowed
5. Do not want to disclose

17. What is your employment status? (*Please select one that applies to you*)

1. Full-time (30 or more hours per week)
2. Part-time
3. Contract, Freelance or Temporary Employee
4. Self-employed
5. Semi-retired
6. Retired
7. Homemaker
8. Stay-at-Home Parent
9. Full-time Student
10. Part-time Student (working MORE than 30 hours per week)
11. Part-time Student (working LESS than 30 hours per week)
12. Unemployed
13. Other (please specify)
14. Do not want to disclose

18. What is your highest educational qualification? (*Please select one that applies to you***)**

1. Postgraduate Education Completed (e.g. Masters)
2. Incomplete Secondary Education (Below GC SE / O Level)
3. Secondary Education Completed (A Level or equivalent)
4. Secondary Education Completed (GCSE / O Level / CSE or equivalent)
5. Vocational or Technical Qualifications Completed (e.g. HND, NVQ)
6. Doctorate, Post-doctorate or equivalent (Higher Degree)
7. Prefer not to answer
8. University Education Completed (First Degree e.g. BA, BSc)
9. Some Vocational or Technical Qualifications
10. Do not want to disclose

19. Could you please estimate the annual income of your household before deducting tax and national insurance (if you receive any benefits include them as income)? (Please select one that applies to you)

1. Less than £10000

2. £10000-19,999

3. £20,000-29,999

4. £30,000-39,999

5. £40,000-49,999

6. £50,000-59,999

7. £60,000-69,999

8. £70,000-79,999

9. £80,000-89,999

10. £90,000-99,999

11. £100,000-149,999

12. £150,000-199,999

13. £200,000-499,999

14. 500,000 or more

96. Prefer not to answer

20. Are you the main income earner in your household? (*Please select one that applies to you*)

- Yes
- No
- Don’t Know

21. How many people (including yourself) live in the household? Please only include people you share budget with. **[Programming instructions: 0 should not be allowed to enter in the box, numbers should be 1 or more to be valid]**

**[OENUM text box]**

22. Have you ever had experience of dementia? (Please select those that applies to you) **[Programming Instructions: Respondents should be allowed to choose multiple answers]**

- One of your family member has/had dementia
- One of your friend has/had dementia
- One of your colleague has/had dementia
- One of your relative has/had dementia
- Others (please specify)
- No

23. Are you a carer or care worker?

1. Yes

2. No

24. Please click the ONE box that best describes your health TODAY **[Programming Instructions: Please show this sentence for each block below]**

**[Programming Instructions: This section should be only in the way euroqol suggests. Follow the link to see what exactly is needed –**

**[PN – show each section on a separate screen]**

**[Programming Instructions: Could you please present each block in individual screens with the current format]**

**1. Mobility**

1. I have no problems in walking about
2. I have slight problems in walking about
3. I have moderate problems in walking about
4. I have severe problems in walking about
5. I am unable to walk about

**2. Self-care**

1. I have no problems in washing or dressing myself
2. I have slight problems in washing or dressing myself
3. I have moderate problems in washing or dressing myself
4. I have severe problems in washing or dressing myself
5. I am unable to wash or dress myself

**3. Usual Activities (**e.g. work, study, house work, family or leisure activities**)**

1. I have no problems doing my usual activities
2. I have slight problems doing my usual activities
3. I have moderate problems doing my usual activities
4. I have severe problems doing my usual activities
5. I am unable to do my usual activities

**4. Pain/Discomfort**

1. I have no pain or discomfort
2. I have slight pain or discomfort
3. I have moderate pain or discomfort
4. I have severe pain or discomfort
5. I have extreme pain or discomfort

**5. Anxiety/Depression**

1. I am not anxious or depressed
2. I am slightly anxious or depressed
3. I am moderately anxious or depressed
4. I am severely anxious or depressed
5. I am extremely anxious or depressed

25. We would like to know how good or bad your health is TODAY.

This scale is numbered from 0 to 100.

100 means the best health you can imagine.

0 means the worst health you can imagine.

Please click on the scale to indicate how your health is TODAY.

Your health today = **[OENUM text box]**

**[Programming Instructions: Please check the demo in the following EQ-5D-5L link to see what is expected]**

[**http://eq-5d-demo.euroqol.org/demo**/](http://eq-5d-demo.euroqol.org/demo/) - **PM to check this.**

[Please change this to as demonstrated in the eq-5d link above, currently it is in a simple slider version]

Should you have any questions related to this survey, please contact Research Now quoting your member ID and survey number.

Thank you for your participation.
